# Mental health follow-up and treatment engagement following suicide risk screening in the Veterans Health Administration

**DOI:** 10.1371/journal.pone.0265474

**Published:** 2022-03-17

**Authors:** Nazanin Bahraini, Daniel J. Reis, Bridget B. Matarazzo, Trisha Hostetter, Christina Wade, Lisa A. Brenner

**Affiliations:** 1 VA Rocky Mountain Mental Illness Research, Education and Clinical Center (MIRECC), Rocky Mountain Regional VA Medical Center, Aurora, CO, United States of America; 2 Department of Psychiatry University of Colorado, Anschutz School of Medicine, Aurora, CO, United States of America; 3 Department of Physical Medicine and Rehabilitation, University of Colorado, Anschutz School of Medicine, Aurora, CO, United States of America; 4 Department of Neurology, University of Colorado, Anschutz School of Medicine, Aurora, CO, United States of America; University of California San Diego School of Medicine, UNITED STATES

## Abstract

**Importance:**

Understanding the extent to which population-level suicide risk screening facilities follow-up and engagement in mental health treatment is important as engaging at-risk individuals in treatment is critical to reducing suicidal behaviors.

**Objective:**

To evaluate mental health follow-up and treatment engagement in the Veterans Health Administration (VHA) following administration of the Columbia-Suicide Severity Rating Scale (C-SSRS) screen, a component of the VHA’s universal suicide risk screening program.

**Design:**

This cross-sectional study used data from VA’s Corporate Data Warehouse.

**Settings:**

140 VHA Medical Centers.

**Participants:**

Patients who completed the C-SSRS screen in ambulatory care between October 1, 2018—September 30, 2020.

**Exposure:**

Standardized suicide risk screening.

**Main outcomes and measures:**

Mental health follow-up (one or more visits within 30 days of C-SSRS screening) and treatment engagement (two or more visits within 90 days of C-SSRS screening) were examined.

**Results:**

97,224 Veterans in Fiscal Year 2019 (FY19) (mean age 51.4 years; 86.8% male; 64.8% white, 22.4% African-American) and 58,693 Veterans in FY20 (mean age 49.6 years; 85.5% male; 63.4% white, 21.9% African-American) received the C-SSRS screen. Across FYs, a positive C-SSRS screen was associated with increased probability of mental health follow-up and treatment engagement. Patients who were not seen in mental health in the year prior to screening had the greatest increase in probability of mental health follow-up and engagement following a positive screen (*P*<0.001). For FY19, a positive C-SSRS screen in non-mental health connected patients was associated with an increased probability of follow-up from 49.8% to 79.5% (relative risk = 1.60) and engagement from 39.5% to 63.6% (relative risk = 1.61). For mental health-connected patients, a positive C-SSRS screen was associated with a smaller increase in probability of follow-up from 75.8% to 87.6% (relative risk = 1.16) and engagement from 63.3% to 76.4% (relative risk = 1.21). Results for FY20 were similar.

**Conclusions and relevance:**

Identification of suicide risk through population-level screening was associated with increased mental health follow-up and engagement, particularly for non-mental health connected patients. Findings support the use of a standardized, comprehensive suicide risk screening program for managing elevated suicide risk in a large healthcare system.

## Introduction

In October of 2018, the Veterans Health Administration (VHA) implemented a national enterprise-wide suicide risk screening and evaluation initiative known as the VA Suicide Risk Identification Strategy (Risk ID) [1]. The goal of Risk ID is to standardize the tools and processes for suicide risk screening and evaluation across healthcare settings. Risk ID also expands suicide risk screening to non-mental health (MH) settings, such as primary care and the Emergency Department (ED), as research has shown that a significant number of people who die by suicide present to these medical settings in the months before their death [2, 3]. As such, Risk ID was designed to maximize detection of suicide risk among patients presenting to routine medical settings and to ensure that those deemed to be at elevated risk receive appropriate follow-up and intervention.

Risk ID facilitates identification of suicide risk through two key processes: suicide risk screening and comprehensive suicide risk evaluation. Early implementation of Risk ID (October 2018- December 2020) consisted of two stages of screening to identify individuals with actionable risk who may require further evaluation: the primary suicide risk screen, item 9 (i9) of the Patient Health Questionnaire-9 (PHQ-9), and the secondary suicide risk screen, the Columbia-Suicide Severity Rating Scale (C-SSRS) screen. [4, 5].

Bahraini et al. [6] demonstrated that implementation of Risk ID was not only feasible in medical settings, but also helped identify patients at risk for suicide who may not be engaged in MH care. During the first year of implementation, more than four million Veterans in ambulatory care settings and more than one million Veterans in the ED underwent primary suicide risk screening as mandated. Furthermore, more than two-thirds of those with a positive primary screen received timely secondary screening using the C-SSRS screen.

While these findings are promising, further research is needed to evaluate the clinical utility of Risk ID. Ideally, a positive screen for suicide risk will facilitate timely connection to behavioral or MH services for further evaluation and/or management of risk as clinically indicated. Understanding the extent to which population-level suicide risk screening facilities follow-up and engagement in MH treatment is important, as research has shown that engaging individuals at risk for suicide in treatment is critical to reducing suicidal behaviors [7–10]. More importantly, most of the research on treatment engagement among at-risk patients has focused on the transition from psychiatric inpatient settings or the ED to outpatient MH services [9–11]. Little research has focused on MH follow-up and treatment engagement following identification of risk in non-acute medical settings. In this study, we examined the extent to which population-level suicide risk screening in non-acute medical settings facilitates MH follow up and treatment for those identified to be at risk. We hypothesized that Veterans identified to be at elevated risk (i.e., positive secondary screen) in non-acute care settings were more likely than those with a negative screen to have a MH follow-up within one month of screening and to engage in at least two MH visits within three months of screening. Given that Risk ID was designed to maximize detection of risk among those who may not currently be engaged in MH treatment, we also hypothesized that the association between a positive secondary screen and likelihood of MH follow up and treatment engagement would be greater among those without a recent history of MH contact compared to those with a recent history of MH contact.

## Methods

This was a nationwide, retrospective study of pre-existing medical records for patients receiving care in the VHA. Additional details regarding Risk ID can be found in Bahraini et al. [6]. This study was exempted from review by the Institutional Review Board and approved by the VA Research and Development Committee. Reporting follows the Strengthening the Reporting of Observational Studies in Epidemiology (STROBE) guideline [12].

### Settings & patients

Data were extracted for patients who: a) were eligible for suicide risk screening during fiscal years (FY) 2019–2020 (October 1, 2018 to September 30, 2020); and, b) completed the C-SSRS screen after a positive primary screen. Patients were eligible for Risk ID screening if they had at least one outpatient visit in an ambulatory care setting (e.g., primary care, cardiology, or MH) and were due for depression and/or PTSD screening at the time of the visit. As part of Risk ID, the primary screen (i9 of the PHQ-9) [4] was added to the depression and PTSD screens. Those who had a positive i9 went on to receive the C-SSRS screen. Those with a positive C-SSRS Screen were required to receive a same day comprehensive suicide risk evaluation. The evaluation could be conducted by any licensed independent practitioner and did not require a mental health referral.

### Measures

The C-SSRS screen is a 6-item measure derived from the original version of the C-SSRS [5]. The first five items of the C-SSRS screen assess severity of suicidal ideation, including method, intent, and plan; the final item, comprised of two parts, asks about lifetime and recent suicidal behaviors. All items are answered with “yes” or “no.” A positive C-SSRS screen was defined as a “yes” response to items 3, 4, 5, or 6b. Previous studies have demonstrated the reliability and validity of the original and screen versions of the C-SSRS [5, 13].

### Data sources

Data were extracted from the VA Corporate Data Warehouse. Dates of MH visits—defined as VHA outpatient encounters that used a 500-series Decision Support System Identifier (“stop code”) in the primary or secondary position and counted for workload credit—were used to generate the two primary outcomes for this study: MH follow-up and MH engagement. These stop codes included all MH specialty (e.g., homeless, substance use, trauma, severe mental illness) and integrated care clinics (Primary Care Mental Health Integration). MH follow-up was defined as having at least one MH visit within 30 days of C-SSRS screening, and MH engagement was defined as having at least two MH visits, on separate days, within 90 days of C-SSRS screening. Additionally, these dates were used to establish whether a patient had a history of recent VHA MH treatment, which was defined as having at least one MH visit in the year prior to C-SSRS screening.

Demographic information and MH diagnosis history were also extracted for eligible patients. For a given patient, MH-related International Classification of Diseases and Related Health Problems, 10^th^ Revision (ICD-10) codes were obtained from any VHA outpatient encounter in the year prior to screening. The codes were then grouped into the presence or absence of the following five diagnostic categories: substance use disorders, defined as any F10-F19 ICD-10 code (or more specific subcode); mood disorders, including bipolar disorders, defined as any F30-F34 or F39 ICD-10 code; anxiety disorders, defined as any F40-F42 ICD-10 code; posttraumatic stress disorder (PTSD), defined as an F43.1 ICD-10 code; and other mental health-related disorders not otherwise captured in the preceding categories. A sixth domain, marked as “MH diagnoses unknown,” was used with patients for whom no diagnostic information was available because they had zero outpatient encounters that required a diagnosis across the VHA system in the prior year. The decision to create the “MH diagnoses unknown” category was made to permit inclusion of this subgroup of cases in analyses rather than remove them for having missing data and to avoid potentially mischaracterizing this subgroup as having no MH diagnoses (e.g., those with mental illness who had previously been receiving care outside of the VHA). It should be noted that this category likely captures information regarding overall healthcare engagement in addition to MH diagnoses.

### Analysis

Analyses were conducted using R 4.0.5 software [14]. Modified Poisson regressions with robust error variance were used to examine the associations between C-SSRS screening and future MH treatment, as this approach allows for the correct estimation of relative risk when using binary outcomes [15]. Outcome variables were MH follow-up and MH engagement. Predictor variables were C-SSRS screen results, history of MH treatment, and their interaction. Covariates were age, gender, race, ethnicity, and MH diagnosis. Separate regression analyses were estimated for each outcome. Separate analyses were also conducted for each FY because screening often occurs on an annual basis, and screening results could differ for patients across fiscal years. Results are reported as relative risks with 95% confidence intervals. Alpha was set at 0.05.

## Results

Patient demographic information is presented in Table 1. C-SSRS screening results were available for 155,917 patients across the two FYs, with 97,224 and 58,693 unique patients in FY19 and FY20, respectively. The study sample was primarily male, white, and not Hispanic or Latino/a, and middle-aged. Mood disorders and PTSD were the two most common historical diagnostic categories, occurring in approximately one-fourth to one-third of patients. Approximately 18% of C-SSRS screens were positive and around one-half of patients had been seen in MH in the year prior to C-SSRS screening. Overall, 70% of the study sample had MH follow-up and approximately 59% engaged in MH care.

**Table 1:** Sample demographics.

	Total (n = 155,917)	FY2019 (n = 97,224)	FY2020 (n = 58,693)
Positive C-SSRS	28,005 (17.96)	17,000 (17.49)	11,005 (18.75)
Mental health visit in last year	81,365 (52.18)	49,960 (51.39)	31,405 (53.51)
Timely mental health follow-up	109,146 (70.00)	66,001 (67.89)	43,145 (73.51)
Mental health engagement	91,951 (58.97)	55,205 (56.78)	36,746 (62.61)
Age, mean (SD)	50.73 (17.23)	51.4 (17.17)	49.61 (17.26)
Female	21,422 (13.74)	12,913 (13.28)	8,509 (14.5)
**Race**			
Black or African American	34,573 (22.17)	21,743 (22.36)	12,830 (21.86)
American Indian or Alaska Native	1,944 (1.25)	1,211 (1.25)	733 (1.25)
Asian	3,238 (2.08)	1,970 (2.03)	1,268 (2.16)
Native Hawaiian or Other Pacific Islander	2,230 (1.43)	1,351 (1.39)	879 (1.5)
White	100,163 (64.24)	62,960 (64.76)	37,203 (63.39)
Multi-racial	2,248 (1.44)	1,335 (1.37)	913 (1.56)
Race unknown	11,521 (7.39)	6,654 (6.84)	4,867 (8.29)
**Ethnicity**			
Hispanic or Latino/a	16,329 (10.47)	9,978 (10.26)	6,351 (10.82)
Not Hispanic or Latino/a	133,219 (85.44)	83,688 (86.08)	49,531 (84.39)
Ethnicity unknown	6,369 (4.08)	3,558 (3.66)	2,811 (4.79)
**Psychiatric diagnoses** ^**a**^			
No disorder	48,185 (30.9)	30,740 (31.62)	17,445 (29.72)
Mood disorder	28,480 (18.27)	17,393 (17.89)	11,087 (18.89)
Substance use disorder	46,551 (29.86)	28,331 (29.14)	18,220 (31.04)
Anxiety disorder	26,893 (17.25)	16,076 (16.54)	10,817 (18.43)
PTSD	41,608 (26.69)	25,267 (25.99)	16,341 (27.84)
Other psychiatric disorder	34,220 (21.95)	20,933 (21.53)	13,287 (22.64)
Diagnoses unknown^b^	21,991 (14.1)	13,719 (14.11)	8,272 (14.09)

Note: Values are presented as counts (%) unless otherwise noted.

^a^Diagnoses are not mutually exclusive and will not sum to 100%.

^b^Diagnostic information was unavailable because these patients did not have any outpatient visits that required a diagnosis (across entire VHA) in the year prior to screening.

**Fig 1:**
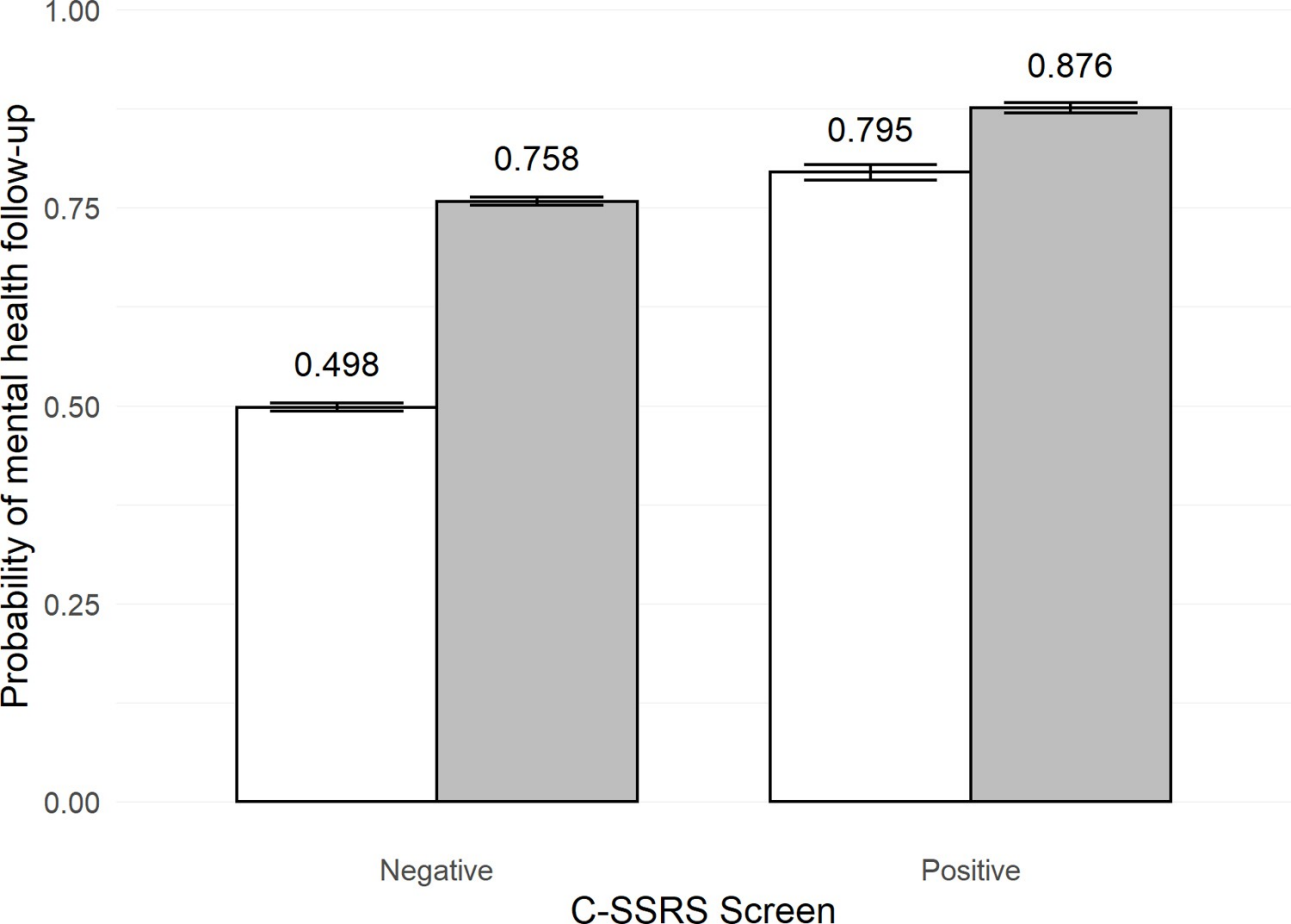
Probability of mental health follow-up after C-SSRS screening in fiscal year 2019. The gray bars represent having received mental health treatment in past year. The white bars represent not having received mental health treatment in the past year. Error bars represent standard error. C-SSRS = Columbia-Suicide Severity Rating Scale.

Results for MH follow-up are presented in Table 2. Across FYs, a positive C-SSRS screen and a recent history of MH contact were each associated with increased probability of MH follow-up. Statistically significant interactions between these two variables were also detected in both years, resulting in a *greater* increase in probability of follow-up for patients without recent MH contact who screened positive versus patients who screened positive but had a MH visit in the past year. This effect was quite substantial (Fig 1). For FY19, a positive C-SSRS screen in patients without recent MH contact was associated with an increase in probability of follow-up from 49.8% to 79.5% (relative risk = 1.60). For MH-connected patients a positive screen was associated with a smaller increase in probability of follow-up from 75.8% to 87.6% (relative risk = 1.16). Results for FY20 were similar, albeit with a slightly higher probability of MH follow-up across all conditions (see S1 Fig).

**Table 2:** Timely mental health follow-up (1+ visits within 30 days of screen).

	Fiscal year 2019						Fiscal year 2020					
	Est	SE	RR	RR 95% CI	*Z*	*p*	Est	SE	RR	RR 95% CI	*Z*	*p*
Intercept	-0.80	0.01	0.45	[0.44, 0.45]	-129.33	< .001*	-0.64	0.01	0.53	[0.52, 0.53]	-90.33	< .001*
C-SSRS+	0.47	0.01	1.60	[1.57, 1.62]	64.15	< .001*	0.35	0.01	1.42	[1.40, 1.45]	42.66	< .001*
Prior MH	0.42	0.01	1.52	[1.50, 1.54]	57.18	< .001*	0.33	0.01	1.40	[1.37, 1.42]	40.14	< .001*
C-SSRS+ * Prior MH	-0.32	0.01	0.72	[0.71, 0.74]	-38.70	< .001*	-0.22	0.01	0.80	[0.79, 0.81]	-23.62	< .001*
Age (scaled)	-0.13	0.00	0.88	[0.88, 0.89]	-54.58	< .001*	-0.11	0.00	0.90	[0.89, 0.90]	-41.36	< .001*
Female (ref: Male)	0.01	0.01	1.01	[1.00, 1.03]	2.62	.009*	0.02	0.01	1.02	[1.01, 1.03]	2.91	.004*
Race (ref: White)												
Black or African American	0.08	0.00	1.08	[1.07, 1.09]	15.99	< .001*	0.06	0.01	1.06	[1.05, 1.07]	11.03	< .001*
American Indian or Alaska Native	-0.04	0.02	0.96	[0.93, 1.00]	-1.89	.059	-0.01	0.02	0.99	[0.95, 1.03]	-0.51	.612
Asian	0.01	0.01	1.01	[0.98, 1.04]	0.59	.555	-0.01	0.02	0.99	[0.96, 1.02]	-0.52	.604
Native Hawaiian or Other Pacific Islander	0.04	0.02	1.04	[1.01, 1.08]	2.43	.015*	0.01	0.02	1.01	[0.97, 1.05]	0.36	.718
Multi-racial	0.02	0.02	1.02	[0.99, 1.06]	1.52	.130	0.04	0.02	1.04	[1.01, 1.07]	2.41	.016*
Race unknown	-0.01	0.01	0.99	[0.97, 1.01]	-1.02	.309	0.00	0.01	1.00	[0.99, 1.02]	0.41	.685
Ethnicity (ref: not Hispanic or Latino/a)												
Hispanic or Latino/a	0.02	0.01	1.02	[1.01, 1.03]	2.78	.005*	0.04	0.01	1.04	[1.02, 1.05]	5.27	< .001*
Ethnicity unknown	0.00	0.01	1.00	[0.98, 1.02]	-0.05	.961	0.00	0.01	1.00	[0.98, 1.03]	0.36	.718
Diagnoses (ref: no diagnosis)^a^												
SUD	0.05	0.00	1.05	[1.04, 1.06]	9.84	< .001*	0.05	0.01	1.05	[1.04, 1.06]	8.65	< .001*
Mood disorder	0.07	0.00	1.08	[1.07, 1.09]	15.90	< .001*	0.04	0.01	1.04	[1.03, 1.05]	7.91	< .001*
Anxiety disorder	0.02	0.00	1.02	[1.01, 1.03]	3.47	.001*	0.02	0.01	1.02	[1.01, 1.03]	4.33	< .001*
PTSD	0.04	0.00	1.04	[1.03, 1.05]	8.00	< .001*	0.02	0.01	1.02	[1.01, 1.03]	3.41	.001*
Other disorder	0.04	0.00	1.04	[1.03, 1.05]	8.84	< .001*	0.03	0.01	1.03	[1.02, 1.04]	5.02	< .001*
Diagnoses unknown	0.23	0.01	1.26	[1.25, 1.28]	29.89	< .001*	0.19	0.01	1.21	[1.19, 1.23]	21.27	< .001*

Note: SE calculated via sandwich estimation, which was used for significance testing. C-SSRS+, positive Columbia-Suicide Severity Rating Scale screen; Est, estimate; Prior MH, Mental health visit in past year; PTSD, Posttraumatic stress disorder; RR, Relative Risk; SE, Standard error; SUD, Substance use disorder.

^a^Diagnoses are not mutually exclusive.

Several covariates also had statistically significant associations with MH follow-up, though the magnitude of relative risk for most of these tended to be fairly small. Female gender was associated with increased probability of MH follow-up in both years, as was Hispanic or Latino/a ethnicity. Compared to White Veterans, African American Veterans were more likely to receive MH follow-up across both years and Native Hawaiian or Other Pacific Islander Veterans were more likely to receive MH follow-up in FY19 only. The two covariates with the largest relative risks were age and having unknown MH diagnostic information. Older Veterans were less likely to receive MH follow-up, and Veterans without available diagnostic information (i.e., those who were not seen in VHA outpatient care in the previous year) had an increased probability of follow-up compared to Veterans without documented MH diagnoses in the year prior to screening.

**Fig 2:**
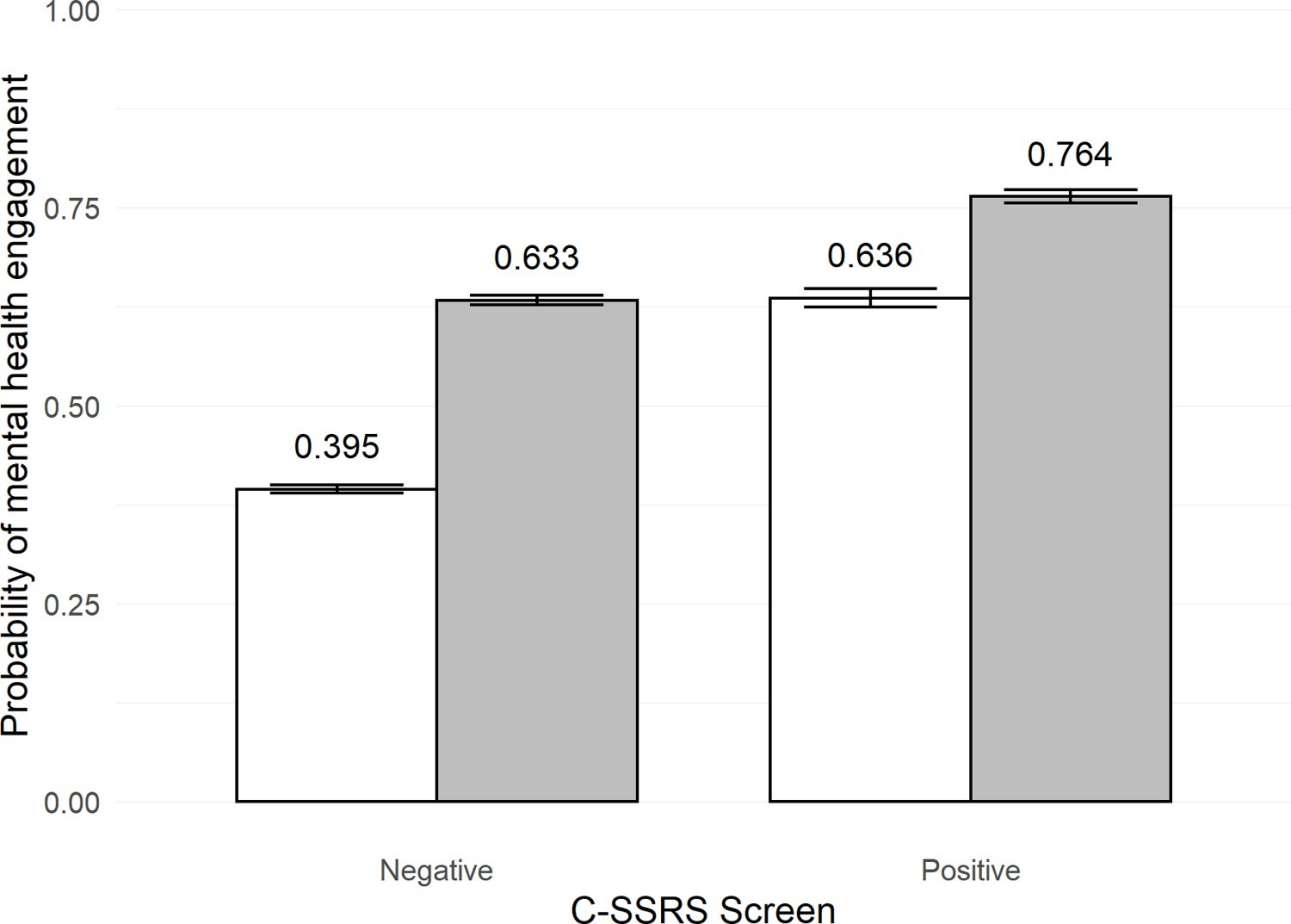
Probability of mental health engagement after C-SSRS screening in fiscal year 2019. The gray bars represent having received mental health treatment in past year. The white bars represent not having received mental health treatment in the past year. Error bars represent standard error. C-SSRS = Columbia-Suicide Severity Rating Scale.

Results for MH engagement are presented in Table 3. Similar to MH follow-up, a positive C-SSRS screen and a recent history of MH contact were each associated with increased probability of MH engagement across FYs, and interactions between these variables were statistically significant. The greatest increase in probability of MH engagement following a positive C-SSRS screen was seen in Veterans without recent MH contact (Fig 2). For FY19, a positive C-SSRS screen in patients without recent MH contact was associated with an increase in probability of MH engagement from 39.5% to 63.6% (relative risk = 1.61). For MH-connected patients, a positive screen was associated with a smaller increase in probability of engagement from 63.3% to 76.4% (relative risk = 1.21). Similar results were found for FY20 (see S2 Fig).

**Table 3:** Mental health engagement (2+ visits within 90 days of screen).

	Fiscal year 2019						Fiscal year 2020					
	Est	SE	RR	RR 95% CI	*Z*	*p*	Est	SE	RR	RR 95% CI	*Z*	*p*
Intercept	-0.60	0.01	0.55	[0.54, 0.56]	-48.92	< .001*	-0.49	0.01	0.61	[0.60, 0.63]	-35.61	< .001*
C-SSRS+	0.48	0.01	1.61	[1.58, 1.64]	46.10	< .001*	0.37	0.01	1.45	[1.41, 1.48]	31.32	< .001*
Prior MH	0.47	0.01	1.60	[1.58, 1.63]	50.44	< .001*	0.40	0.01	1.48	[1.45, 1.52]	36.77	< .001*
C-SSRS+ * Prior MH	-0.29	0.01	0.75	[0.73, 0.77]	-24.49	< .001*	-0.20	0.01	0.82	[0.80, 0.84]	-14.55	< .001*
Age (scaled)	-0.01	0.00	0.99	[0.99, 0.99]	-53.89	< .001*	-0.01	0.00	0.99	[0.99, 0.99]	-42.66	< .001*
Female (ref: Male)	0.05	0.01	1.05	[1.04, 1.07]	7.57	< .001*	0.04	0.01	1.04	[1.02, 1.06]	5.05	< .001*
Race (ref: White)												
Black or African American	0.07	0.01	1.07	[1.06, 1.09]	11.50	< .001*	0.07	0.01	1.07	[1.06, 1.09]	9.80	< .001*
American Indian or Alaska Native	-0.06	0.02	0.94	[0.89, 0.98]	-2.60	.009*	-0.05	0.03	0.95	[0.90, 1.00]	-1.87	.061
Asian	-0.07	0.02	0.94	[0.90, 0.97]	-3.29	.001*	-0.06	0.02	0.94	[0.90, 0.99]	-2.59	.010*
Native Hawaiian or Other Pacific Islander	0.00	0.02	1.00	[0.96, 1.05]	0.07	.948	0.01	0.02	1.01	[0.97, 1.07]	0.60	.550
Multi-racial	0.00	0.02	1.00	[0.96, 1.04]	-0.15	.881	0.06	0.02	1.06	[1.02, 1.10]	2.69	.007*
Race unknown	-0.04	0.01	0.96	[0.94, 0.99]	-2.96	.003*	0.02	0.01	1.02	[1.00, 1.04]	1.60	.111
Ethnicity (ref: not Hispanic or Latino/a)												
Hispanic or Latino/a	0.02	0.01	1.02	[1.01, 1.04]	2.90	.004*	0.06	0.01	1.06	[1.05, 1.08]	6.78	< .001*
Ethnicity unknown	-0.01	0.02	0.99	[0.96, 1.02]	-0.89	.375	0.00	0.02	1.00	[0.97, 1.03]	0.09	.928
Diagnoses (ref: no diagnosis)^a^												
SUD	0.10	0.01	1.10	[1.09, 1.11]	16.18	< .001*	0.08	0.01	1.09	[1.07, 1.10]	12.04	< .001*
Mood disorder	0.12	0.01	1.12	[1.11, 1.14]	19.07	< .001*	0.10	0.01	1.10	[1.09, 1.12]	13.84	< .001*
Anxiety disorder	0.03	0.01	1.03	[1.02, 1.04]	4.79	< .001*	0.04	0.01	1.04	[1.02, 1.05]	5.01	< .001*
PTSD	0.04	0.01	1.04	[1.03, 1.06]	7.43	< .001*	0.02	0.01	1.02	[1.01, 1.03]	3.16	.002*
Other disorder	0.08	0.01	1.08	[1.07, 1.09]	13.04	< .001*	0.05	0.01	1.05	[1.04, 1.07]	7.67	< .001*
Diagnoses unknown	0.29	0.01	1.33	[1.30, 1.36]	27.89	< .001*	0.25	0.01	1.28	[1.25, 1.31]	21.45	< .001*

Note: SE was calculated via sandwich estimation, which was used for significance testing. C-SSRS+, positive Columbia-Suicide Severity Rating Scale screen; Est, estimate in log-relative risk scale; Prior MH, Mental health visit in past year; PTSD, Posttraumatic stress disorder; RR, Relative Risk; SE, Standard error; SUD, Substance use disorder.

^a^Diagnoses are not mutually exclusive.

Again, the relative risks for statistically significant covariates tended to be fairly small, and Veterans without available MH diagnostic information were more likely to engage with MH compared to Veterans without documented MH diagnoses in the year prior to screening. Female gender was associated with increased probability of MH engagement in both years, as was Hispanic or Latino/a ethnicity. Compared to White Veterans, African American Veterans were more likely to have MH engagement in FY19, whereas American Indian or Alaska Native Veterans, Asian Veterans and Veterans for whom race was unknown were less likely. In FY20, compared to White Veterans, African American Veterans and Multi-racial Veterans were more likely to have MH engagement, whereas Asian Veterans were less likely.

## Discussion

To our knowledge, this study is the first to show that population-based suicide risk screening in non-acute care settings facilities MH follow-up and treatment engagement among those identified to be at elevated risk. Overall, findings demonstrated that among patients screened in VHA ambulatory care settings, the probability of MH follow-up and longer-term engagement was higher among those who had a positive secondary screen for suicide risk, compared to those with a negative secondary screen.

Approximately half of the Veterans who received the C-SSRS screen as part of secondary level screening did not have a VHA MH encounter in the year prior to screening and a subset of these individuals did not have *any* outpatient VHA encounter in the year prior to screening. Yet, all of these patients reported some level of suicidal ideation on the primary suicide screen (i.e., i9 of the PHQ-9 measure [4]) which triggered administration of the C-SSRS screen. Notably, patients *without* recent connection to MH who screened positive on the C-SSRS screen had the *greatest* increase in probability of both being seen by MH within one month and attending multiple visits within three months. In FY19, unconnected patients who screened positive on the C-SSRS screen were 60% more likely to have a MH follow-up visit and 61% more likely to engage in MH care compared to those who screened negative. On the other hand, MH-connected patients who screened positive were only 16% more likely to have a MH follow-up visit and 21% more likely to engage in MH care compared to their negatively screened counterparts.

These data suggest that population-level suicide risk screening may be an effective method of not only identifying patients with elevated yet likely unrecognized risk, but also engaging these patients in MH care. The findings for non-MH connected patients are particularly compelling given that most of the studies in this area have primarily focused on outpatient treatment engagement following acute care (e.g., ED) [8–10].

Even among MH-connected patients, those with a positive C-SSRS screen were still 16% more likely to have timely follow-up compared to those with a negative screen. Given that most of these patients had timely follow-up regardless of screening results, even a 16% increase associated with a positive C-SSRS screen is clinically meaningful. For a subset of these patients, suicide risk screening may help facilitate timely re-engagement in MH care or it may prompt more targeted interventions aimed at reducing suicide risk.

Lower probability of engagement, compared to follow-up, after a positive suicide screen were shown in both FY19 and FY20. There are several likely explanations for this finding. It may be that providers are prioritizing quick connection following a positive secondary screen for further suicide risk evaluation. Once evaluated, patients who are deemed to be at higher levels of risk may continue engaging in MH treatment over a longer period of time, while those at lower levels of acute risk may be managed in primary care or through other brief interventions (e.g., safety planning). However, as demonstrated in other studies, both patient level barriers (e.g., attitudes about MH care, perceived need for MH care, availability of other resources, lack of readiness to change), and provider/systems level barriers (e.g., limited clinician availability, environment of care, limited understanding or experience working with high-risk patients, and provider attitudes towards suicide) could have also contributed to lower probability of ongoing MH engagement [16, 17]. Future research is needed to examine specific barriers and facilitators to MH follow-up and engagement after a positive suicide risk screen and how these may differ between patients with and without a history of MH treatment.

Although results across fiscal years were not statistically compared, increased probabilities of follow-up and engagement were observed in FY20. There were also fewer C-SSRS screens completed in FY20 due to the overall decrease in healthcare visits and encounters following the onset of the coronavirus disease of 2019 (COVID-19) pandemic. Despite the decrease in encounters, it is notable that probabilities of MH follow-up and engagement following a positive screen increased in FY20. The shift to system-wide virtual care brought on by the pandemic may have facilitated increased MH engagement by reducing certain barriers to care. Veterans at risk for suicide may also have been more motivated to engage in MH treatment due to the additional stressors brought on by the pandemic (e.g., limited contact with social supports, changes in employment). Though we did not specifically examine pre- and post-COVID-19 changes in screening and follow-up during FY20, further research examining the impact of COVID-19 on the implementation of suicide risk screening, the prevalence of positive suicide screens and follow-up care may shed further light on these findings.

There are several potential limitations with this study. MH encounters were determined using the 500 series stop codes which may have led to instances in which MH encounters were missed because they were classified incorrectly or MH care was given in other integrative care settings not captured in the 500 series (e.g., Rehabilitation Medicine, Home Based Primary Care, Caregiver Support). Another limitation is that we examined any MH treatment encounter in 30- and 90-days post-screening and did not differentiate between the type of encounter, location of care, or procedures received. Future research examining the characteristics and delivery of MH care following a positive suicide risk screen may help inform strategies to increase treatment engagement among at-risk patients. In particular, examining differences in follow-up and engagement across MH specialty and integrated care clinics (e.g., Primary Care- Mental Health Integration) is one area that warrants further investigation and may provide a better understanding of whether these models of care are differentially associated with ongoing treatment engagement for MH-connected and non-MH connected patients. Furthermore, it will also be important to understand which practices may be more effective at engaging patients at different levels of acute and chronic risk (i.e., intermediate, or high) in MH care. Lastly, future research should also focus on examining the impact of suicide risk screening and subsequent care processes on suicide outcomes.

## Conclusions

Connecting and engaging at-risk patients in MH care is a cornerstone of suicide prevention. This study demonstrated that a nationwide population-level suicide risk screening program facilitated follow-up MH care and longer-term MH treatment engagement among those identified to be at elevated risk. The probability of timely MH follow-up and treatment engagement was even higher among patients who were not connected to MH in the year prior to screening. Future research examining factors that impact MH treatment engagement following a positive suicide screen in medical settings is needed to ensure that patients with elevated risk are connected to needed treatment. Moreover, in order to fully understand the impact of population-level screening on suicide, studies that examine the degree to which screening facilitates access to timely evidence-based interventions shown to reduce suicidal behavior are also warranted.

## Supporting information

S1 Fig Fiscal year 2020 follow-up. The gray bars represent having received mental health treatment in past year. The white bars represent not having received mental health treatment in the past year. Error bars represent standard error. C-SSRS = Columbia-Suicide Severity Rating Scale.
(PDF)

S2 Fig Fiscal year 2020 engagement. The gray bars represent having received mental health treatment in past year. The white bars represent not having received mental health treatment in the past year. Error bars represent standard error. C-SSRS = Columbia-Suicide Severity Rating Scale.
(PDF)

S1 Appendix STROBE checklist. (DOCX)
