# Supplementary material for: Mental health follow-up and treatment engagement following suicide risk screening in the Veterans Health Administration
Source: PLoS One. 2022 Mar 17;17(3):e0265474. doi: 10.1371/journal.pone.0265474 (PMC8929551; doi:10.1371/journal.pone.0265474)
Supplement: S2 Fig — The gray bars represent having received mental health treatment in past year. The white bars represent not having received mental health treatment in the past year. Error bars represent standard error. C-SSRS = Columbia-Suicide Severity Rating Scale. (PDF) [file pone.0265474.s002.pdf]

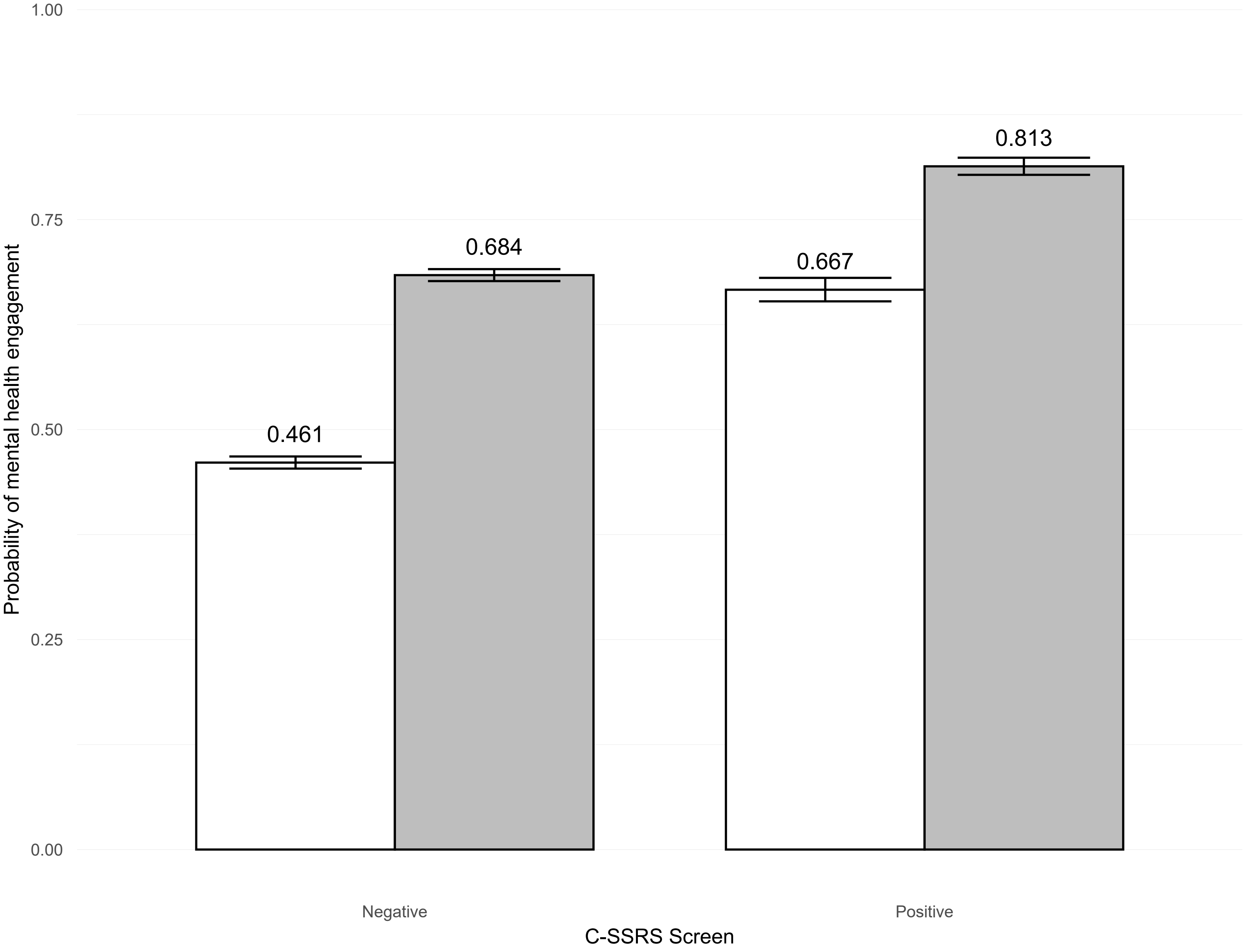

S2 Figure. Fiscal Year 2020 Engagement. White bars = No mental health treatment in past year. Gray bars = Mental health treatment in past year.
